# Rapamycin in mice

**DOI:** 10.18632/aging.101289

**Published:** 2017-09-01

**Authors:** William R. Swindell

**Affiliations:** Ohio University, Heritage College of Osteopathic Medicine, Athens, OH, 45701 USA

**Keywords:** healthy aging, lifespan, longevity, meta-analysis, mTOR, NNT, survivorship

The United States Preventative Services Task Force (USPSTF) was established in 1984 to provide evidence-based guidelines for delivery of preventative health care services. The USPSTF goal has been to improve clinical outcomes by leveraging the advantages of preventative care, with emphasis on preventing disease rather than merely treating it. At the time USPSTF was established, a vigorous effort was underway to identify aging biomarkers [1], which would in principle lay groundwork for future studies into basic aging mechanisms and, potentially, assist with the identification of interventions influencing these mechanisms. In many ways, these long-term goals of basic aging research were complementary and consistent with those of USPSTF, since the concept of “prevention” is fundamental to the rationale underlying development of drugs targeting basic aging mechanisms. Although *bona fide* “anti-aging” drugs have not yet achieved clinical validation in humans, important steps forward have been made, with one human trial now underway to evaluate efficacy of metformin in healthy glucose-tolerant subjects [2].

The metformin study is scheduled for completion in late 2017 and will provide an important milestone for clinical translation in aging research [2]. However, based on existing animal data, rapamycin (Sirolimus) remains the most promising drug with the potential to alter the preventative medicine landscape [3]. Rapamycin is an mTOR inhibitor that binds FK506-binding protein (FKBP) and prevents IL-2 responses by blocking T-cell activation and B-cell differentiation. The drug is now used clinically for rejection prophylaxis in kidney transplant patients and is also used in drug-eluting stents to prevent restenosis. Few studies, however, have been performed to evaluate use of rapamycin for disease prevention in asymptomatic adults, although one study suggested that mTOR inhibition bolsters vaccine responses in elderly subjects [4].

Pre-clinical evidence supporting beneficial effects of rapamycin on lifespan has been buoyed by mouse survivorship studies [3], which may be concerning since prior work has shown that results from such studies can vary among laboratories and be influenced by strain or other factors [5]. This complicates their interpretation, since for example it is unclear which mouse strains provide the best models for translation to human populations [5]. To explore the role of strain and other factors, a meta-analysis of 29 mouse survivorship studies was recently carried out [6]. Findings demonstrated significant heterogeneity among studies, but overall meta-estimates were consistent with a 13% lifespan increase in rapamycin-treated mice (95% CI: 11.5% - 14.6%), with stronger increases in females (15.1%) compared to males (9.4%) and a trend towards weaker survivorship increases in pure inbred strains such as C57BL/6 [6]. These results confirm generally favorable effects of rapamycin treatment on mouse lifespan, but also identify sex- and strain-dependent effects influencing the treatment response magnitude.

**Figure 1:**
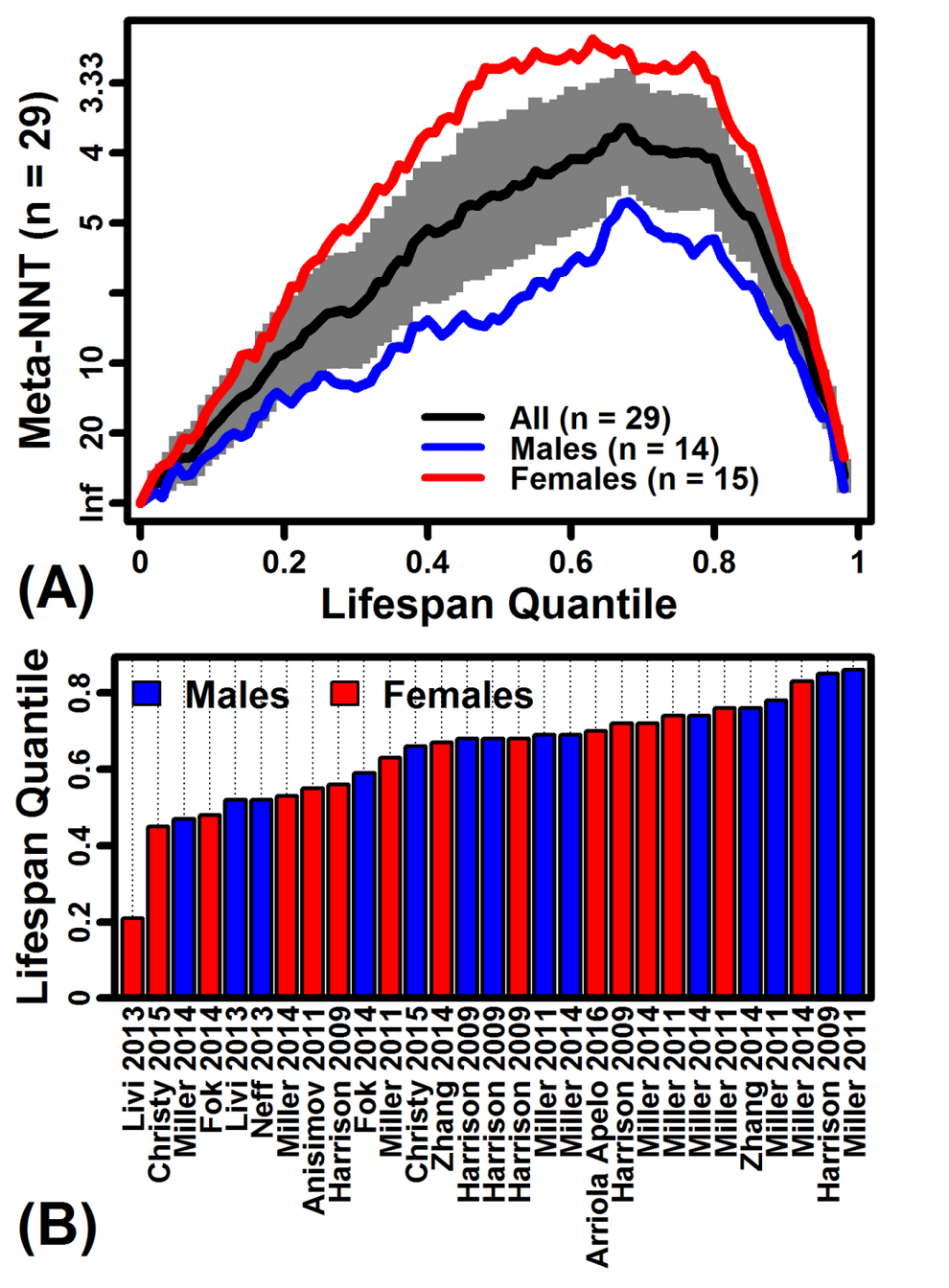
Number needed to treat (NNT) estimates from rapamycin-treated mouse cohorts (**A**) NNT meta-estimates across lifespan quantiles (*n* = 29 survivorship experiments). Survival times in each experiment were converted to quantiles to permit integration across experiments. Absolute risk reductions (ARR) were calculated with respect to each lifespan quantile in each experiment, and then ARR estimates were integrated across studies using a random effects meta-analysis model [6]. The grey region denotes the 95% confidence interval associated with the NNT meta-estimate (black line). (**B**) Lifespan quantile associated with the minimum NNT estimate in each of 29 experiments. Horizontal axis labels are consistent with those used by Swindell [6].

Rapamycin increased mouse lifespan by 13% on average, but this *relative* measure of treatment effect does not provide the complete story and alternative metrics may be more informative from a preventative medicine standpoint [6]. In particular, the age-specific *absolute* survivorship increase may be a more informative measure of treatment effect, since this value is reciprocally related to the number of individuals requiring treatment to yield one additional survivor at a given age (i.e., number needed to treat, NNT). The above-mentioned meta-analysis of 29 experiments estimated a minimal NNT value of 3.10 (95% CI: 2.75–3.55), with the minimum NNT meta-estimate occurring at the 67th percentile survival time and NNT increasing precipitously at later ages (Figure 1A). Among the 29 experiments, the minimum NNT always occurred prior to the 90th percentile survival time (range: 21st – 86th percentile), reflecting diminution of absolute risk reduction with increasing age (Figure 1B) [6]. In some ways, these patterns challenge the focus of many aging researchers on maximum lifespan extension, instead suggesting that the greatest benefits of preventative rapamycin treatment may be to generate more “rectangular” survival curves by preventing early death, thereby promoting survival to late ages. Numerous factors complicate comparisons between mouse and human NNT estimates, but to provide perspective and context, it can be noted that the rapamycin NNT meta-estimate of 3.10 compares favorably to preventative treatments now used routinely – for example, an NNT of 83 was calculated for mortality prevention by HMG-CoA reductase inhibitors (“statins”) in those with heart disease [7].

Mouse longevity experiments are challenging to perform, expensive, and time-consuming. While survival trends alone do not necessarily provide insights into healthspan, longevity studies will likely continue to be influential within the aging research community. A literature search, for example, reveals that fewer than 10 published papers per year were related to the topic “rapamycin and aging” between 1975 and 2008; however, from 2009 to 2016, approximately 180 papers per year related to this topic (Web of Science database). This 18-fold expansion of research effort attests to the influence of mouse longevity studies. Accordingly, meta-analysis of mouse longevity data is necessary to ensure that research efforts are guided by the most robust conclusions possible [5, 6]. Basic trends from mouse survival data can often be discerned from descriptive analyses alone, but more nuanced conclusions can be obtained through alternative analysis methods yielding both relative and absolute treatment effect measures [6]. Most basic aging research has emphasized relative measures (e.g., hazard ratios), but absolute measures such as NNT may provide a more natural fit within the preventative medicine paradigm. Along these lines, NNT estimates from mouse longevity studies strengthen rapamycin's case as a candidate preventative therapy, although integration with adverse event frequency data will ultimately be needed to fully delineate the cost-benefit profile.
